# The interest to expand group-based studies with single-case experimental design studies in the investigation of the effects of physical exercise in frail older adults: an opinion article

**DOI:** 10.3389/fpubh.2023.1256645

**Published:** 2023-11-03

**Authors:** Nounagnon Frutueux Agbangla, Isabelle Caby, Cédric T. Albinet

**Affiliations:** ^1^Univ. Artois, Univ. Lille, Univ. Littoral Côte d'Opale, ULR 7369 - URePSSS - Unité de Recherche Pluridisciplinaire Sport Santé Société, Liévin, France; ^2^Laboratoire Sciences de la Cognition, Technologie, Ergonomie (SCoTE-EA7420), Université de Toulouse, INU Champollion, Albi, France

**Keywords:** nursing homes, long-term care homes, SCED, physical activity, *frailty*, aging

## Introduction

Human population aging is among the most important transformations of the 21st century. The number of older adults will increase significantly in the coming decades (1). While some older adults will experience successful aging that allows them to be physically, cognitively, and socially active (2), others will develop a pathological aging process that could make them frail. “*Frailty is a condition in which the individual is in a vulnerable state at increased risk of adverse health outcomes and/or dying when exposed to a stressor […] Frailty is either physical or psychological or a combination of the 2 components and is a dynamic condition that can improve or worsen over time”* (3). The care of frail older adults has become a public health and policy priority, as shown by the report of the French Economic, Social, and Environmental Council (4). This care, particularly for older adults with special needs living in institutions, involves several interventions: non-medicinal interventions grouped into psychological interventions (art therapy, health education, psychotherapy, zootherapy), physical interventions (physical activity, physiotherapy, manual therapy, thermalism), nutritional interventions (food supplements, nutritional therapy), digital interventions (connected object, video game therapy, virtual reality therapy), and other more or less scientifically validated interventions (lithotherapy, mycotherapy, ergonomic adjustments, phytotherapy, wave therapy, cosmetic therapy, among others) (5). Concerning physical interventions, the synthesis of work in this population shows the beneficial effect of exercise on mental health, cognition (6), and physical health (7). However, an in-depth review of the reported studies examining exercise effects on frail older adults in care settings reveals two major limitations, as outlined in the following section.

## Limitations of group-based studies and interest of single-case experimental design in investigating the effects of physical exercise on frail older adults

The first limitation is a lack of individualization of the exercise program in terms of intensity, the type of physical activity, duration, and frequency. For methodological reasons, the exercise programs in different studies have been standardized and offered to older adults participating in the programs. However, older adults in care settings are confronted with varied problems (osteoarticular, sensorimotor, sensory, cognitive, and emotional) and present very different profiles. This difference in profiles should be considered and should not lead to the exclusion of some older adults through inclusion or exclusion criteria. For instance, as recently reported by Brach et al. (8), research in this field is typically biased toward healthy and relatively young older adults, with often the exclusion of individuals as a function of chronological age, particular pathologies or disabilities or even social barriers. The second limitation concerns the experimental design used in most studies in the literature. Most research work in this field consists of randomized controlled, cross-sectional, or longitudinal studies, which are group-based methodologies (9–11). The group-based methodologies focus on the average (or median) of the statistically or empirically constructed groups to perform inferential analyses. This is well-justified to draw conclusions about the effects of physical exercise and to generalize them. However, while group-based methodologies have statistical and methodological strengths that allow reliable and useful conclusions to be drawn for the care of frail older adults, they mask the specificities of each single older individual. In addition, the average person presented in the results does not exist and cannot be equated with all participants or the rest of the population that did not participate in the studies (12). Moreover, one must acknowledge that it appears paradoxical to seek individualization of health care and to use group-based treatments to draw conclusions applicable to each individual. Finally, implementing a group-based interventional study with frail older adults in care settings is often complicated, yet sometimes unfeasible, by methodological, ethical, and practical considerations. Actual randomization beyond participants' wishes, the constitution of experimental and control groups, and individual compliance rates to the programs are some of the problems faced by researchers in this field, which sometimes compromise the quality and validity of group-based studies.

To optimize the effects of physical exercise on older adults in care settings, individualization is often implemented by professionals based on the needs and capabilities of frail older adults. Individualization consists in proposing a physical practice by considering the particularities of each person in terms of needs, abilities, and desires (13). However, once the program has been individualized, it remains difficult to draw causal links of individualized intervention programs. For this reason, the Single-Case Experimental Design (SCED) can be used. SCED is defined as “*designs that are applied to experiments in which one entity is observed repeatedly during a certain period of time under different levels of at least one independent variable*” [(12), p. 3]. It differs from the case study, where “*single entity is studied intensively, but there is not necessarily a purpose manipulation of an independent variable, nor are there necessarily repeated measures*” [(12), p. 3]. During SCED, comparison between various experimental phases (baseline, treatment, and follow-up phases) allows us to explore a causal or functional relationship between an independent variable and a significant change in the dependent variable (12, 14). According to Tate et al., four types of SCED can be encountered such as withdrawal/reversal design, multiple baseline design, changing design, and alternating treatment design (15). In withdrawal/reversal, an intervention is applied and withdrawn sequentially, whereas multiple baseline design comprises several baselines and allows sequential application of an intervention, which is also introduced in a staggered manner for a specific parameter. For changing design, several hierarchical criteria levels are established and implemented sequentially. Lastly, alternating treatment design enables us to compare several interventions concomitantly, by alternating the application of the interventions (15). From a methodological point of view, each type of SCED demonstrates its strengths and weaknesses. In terms of strengths, the withdrawal/reversal design enables systematic intra-subject replication by including several treatment phases. With respect to the multiple baseline design, it is found to be robust against the adverse effect of non-controllable variables on internal validity. The alternating treatment design and the changing-criterion design avoid the ethical problem of interrupting effective interventions in the withdrawal/reversal design (16). Finally, SCEDs put the individual at the center of the protocol, reduce the gap between researchers and participants, and provide immediate feedback during the intervention to ensure that adjustments can be made if necessary (12). They appear thus particularly appropriate to evaluate the potential effects of individualized programs in care settings. Despite these strengths, each SCED may present specific weaknesses. Disadvantageous effects during withdrawal/reversal design and alternating treatment design include sequential confounding, carryover, and alternation. Indeed, the sequential confounding effect refers to the influence that the order of introducing the interventions may have on the efficacy of one or both interventions. The carryover effect is the influence of one treatment on an adjacent treatment, whatever the influence of the overall sequence. Finally, the alternation effect occurs because the time between interventions is short (16). Concerning the multiple baseline design, repeated measurement of the target behavior over a long baseline period can be difficult and may result in participant reactivity, but draw the participant's attention to an erroneous performance model (16). Finally, the changing-criterion approach applies to a relatively small range of situations (16), such as behavioral problems in autism for example. Furthermore, SCEDs can pose several difficulties for researchers, such as establishing a representative reference base, managing the non-independence of sequential observations, interpreting the effect size of a single subject, and multiplying measurements (at least 3) during each phase. Finally, SCEDs appear appropriate for research questions and contexts where wider generalization is not the main objective. Considering these mentioned strengths and weaknesses, the SCED may present valuable interests in the field of exercise sciences in the frail population (14). These include the effective study of the individual in the true sense of the word, the control of the practical modalities of an intervention, and the possibility to make necessary adjustments based on continuously collected data (12). In sum, the individualization of the exercise program and the use of the SCED to test its effects in each individual or a homogeneous small group of individuals, with reference to well-defined characteristics, could counteract the principal limitations that we have raised above concerning the group-based designs. Moreover, a new meta-analysis methodology of SCED data has been recently proposed (17) that should stimulate the development of such studies in this field. Nevertheless, the SCED is not exempt from methodological limitations and progress should continue to better increase the quality of experimental protocols, as well as data analyses and study reporting, following recommendations and guidelines (14, 15, 18–20).

Literature reports a tentative use of SCED in frail older adults with dementia and Parkinson's disease (21–25). These studies have tested the effects of cognitive therapies (Psychosocial intervention, Gestalt therapy, Cognitive-behavioral therapy) (21–24) and multimodal therapies (25) on the perceived stress, global cognitive functioning, behavioral and psychological symptoms, depression, anxiety, and independent outdoor activities. One important point is that the results showed that these therapies are efficient for some but not all participants. This kind of findings cannot be reported by group-based studies and calls for developing such SCED studies to better understand qualitatively why and how such programs are beneficial or not to some individuals according to their individual characteristics. However, one must note that, among these reviewed studies, only the study by Yorozuya et al. was conducted in a nursing home and included physical exercises (gymnastics and stretching), which, unfortunately, were not individualized (25). Again, this shows the need to develop more SCED studies to examine the effects of physical exercise in frail older adults.

## Conclusion

To sum up, SCED vis-a-vis frail older adults in care settings is rare. Moreover, the few studies in that domain have not directly tested the effects of an individualized physical exercise program on the physical and mental health of frail older adults. Researchers should mobilize to conduct more SCEDs when appropriate, particularly in care settings where they appear relevant. The multiplication of SCEDs in this research field will enable the supplementation of studies using group-based methodologies to better integrate the quality of individualized care for frail older adults. It will also encourage appreciation of the beneficial therapeutic effects of the intervention (physical exercise) or of individual-level failures, in order to better adjust and regulate the care. SCEDs should target frail older adults suffering from pathologies such as osteoarticular, sensorimotor, sensory, cognitive, and emotional diseases, and those who are typically excluded from group-based studies. This would make it possible to extend existing data on the effects of physical activity in frail older adults.

## Author contributions

NA: Conceptualization, Writing—original draft, Writing—review & editing. IC: Writing—review & editing. CA: Writing—review & editing.
